# The Importance of Radiological Patterns and Small Airway Disease in Long-Term Follow-Up of Postacute COVID-19: A Preliminary Study

**DOI:** 10.1155/2022/7919033

**Published:** 2022-05-05

**Authors:** Roberto Mogami, Ronaldo Carvalho Araújo Filho, Carolina Gianella Cobo Chantong, Fernando Carlos Santos de Almeida, Ana Célia Baptista Koifman, Gustavo Federico Jauregui, Thiago Thomaz Mafort, Hanna da Silva Bessa da Costa, Glenda Aparecida Peres dos Santos, Bruna Zangerolame de Carvalho, Gabriel da Silva Passos, Erick de Souza Barbosa, Angelo Thomaz Abalada Ghetti, Laura Braga Monnerat, Mariana Soares da Cal, Desiree Louise Souza Santos Batista, Helen Aksenow Affonso, Gabriel Oliveira Bousquet, Jose Ignacio Marenco Avila, Anna Luiza Bento Dutra, Caio Leal Leidersnaider, Alexandre Malta da Costa Messeder, Alexandra Monteiro, Agnaldo José Lopes

**Affiliations:** ^1^Department of Radiology, Pedro Ernesto University Hospital, State University of Rio de Janeiro (UERJ), Rio de Janeiro, Brazil; ^2^Medical Sciences Post-Graduation Program, School of Medical Sciences, State University of Rio de Janeiro (UERJ), Rio de Janeiro, Brazil; ^3^Telemedicine and TeleHealth Post-Graduation Program, State University of Rio de Janeiro (UERJ), Rio de Janeiro, Brazil; ^4^Department of Pulmonology, Pedro Ernesto University Hospital, State University of Rio de Janeiro (UERJ), Rio de Janeiro, Brazil

## Abstract

Postacute COVID-19 has become a relevant public health problem, and radiological and pulmonary function tests are tools that help physicians in decision-making. The objectives of this study are to characterize the findings and patterns on a chest radiograph (CXR) and computed tomography (CT) that are most important in the postacute phase and to evaluate how these changes correlate with clinical data, spirometry, and impulse oscillometry (IOS). This was a retrospective study of 29 patients who underwent CXR, CT, spirometry, and IOS. The inclusion criteria were age >18 years and persistent respiratory symptoms after four weeks. The exclusion criteria were radiological exams with low technical quality and non-COVID-19 acute lung diseases. The inferential analysis was carried out with the chi-square (*χ*^2^) or Fisher's exact test to evaluate the interrelationships between the clinical and COVID-19 variables according to spirometry, IOS, CT, and CXR. In our sample, 19 patients were women (65.5%). The predominance of abnormal spirometry was associated with CT's moderate/severe degree of involvement (*p* = 0.017; 69.2%, CI 95%: 44.1%–94.3%). There was no significant association between IOS and tomographic and radiographic parameters. A significant association was found between the classifications of the moderate/severe and normal/mild patterns on CT and CXRs (*p* = 0.003; 93.3%, CI 95%: 77.8%–100%). Patients with moderate/severe impairment on CXR were associated with a higher frequency of hospitalization (*p* = 0.033; 77.8%, CI 95%: 58.6%–97.0%) and had significantly more moderate/severe classifications in the acute phase than the subgroup with normal/mild impairment on CXR (*p* = 0.017; 88.9%, CI 95%: 74.4%–100%). In conclusion, the results of this study show that CXR is a relevant examination and may be used to detect nonspecific alterations during the follow-up of post-COVID-19 patients. Small airway disease is an important finding in postacute COVID-19 syndrome, and we postulate a connection between this pattern and the persistently low-level inflammatory state of the lung.

## 1. Introduction

Sequelae of viral infections that resemble coronavirus disease 2019 (COVID-19) have previously been reported, particularly in outbreaks of severe acute respiratory syndrome (SARS) and the Middle East respiratory syndrome (MERS). A follow-up of MERS patients showed that 36% had residual changes due to fibrosis after six weeks [1].

From a temporal point of view, COVID-19 is considered acute up to four weeks after disease onset, while postacute COVID-19 is the persistence of symptoms or late complications for a period >4 weeks from disease onset, without explanation in terms of diagnosis [2, 3]. This postacute period can be divided into subacute (4–12 weeks) or chronic (over 12 weeks) periods [4, 5]. The most common symptoms in postacute COVID-19 are fatigue, cough, dyspnoea, and neuropsychological disorders [6]. Functional tests show a reduction in the diffusion capacity and a predominance of the obstructive pattern in postacute COVID-19 [1, 7, 8].

The British Thoracic Society recommends collecting chest radiographs for 12 weeks after disease onset during the follow-up of patients with postacute COVID-19. Computed tomography (CT) is necessary to elucidate the diagnosis in cases of persistent disease [9]. Ground-glass opacity (GGO) is the most common abnormality on CT scans. Reticulation and bronchiectasis proportional to the severity of acute disease appeared after three months of follow-up, and the pathophysiology of these changes seems to be related to the sequelae of diffuse alveolar damage and pulmonary thrombosis [10].

D'Cruz et al. [11] studied 119 patients with postacute COVID-19 and found that radiographic abnormalities were present in only 13% of them. On CT, the most common changes were GGO/organizing pneumonia (OP) in 37.5%, small airway disease/bronchiectasis in 16.1%, a combination of interstitial patterns in 8.9%, a combination of interstitial and airway disease in 7.1%, and nonspecific interstitial fibrosis/pneumonia in 5.4%. Of the patients with abnormalities on CT, only 21% also had altered chest radiographs (CXRs).

Wallis et al. [12] analyzed the CXRs of patients with postacute COVID-19 within 12 weeks of development. The CXR tests were abnormal in 32% of the patients. Factors associated with the persistence of radiographic changes were increased age, length of hospital stay, obesity, and increased lactate dehydrogenase levels [12]. The causes of fibrosis in postacute COVID-19 are still not well known but may be related to complications of the hospitalization period, such as mechanical ventilation-induced lung disease induced by mechanical ventilation, hyperoxia, and bacterial pneumonia.

In addition to the findings of GGO and lesions that resemble fibrosis, another relevant data point that is notable, but rarely discussed in the literature, is the involvement of small airways. Few studies have discussed this aspect, but there is a high frequency of air trapping in postacute CT [13–15]. Small airway disease in COVID-19 is likely related to direct compromise of bronchioles or vascular lesions. Immune responses may also be linked to interstitial and bronchial alterations during the postacute period [14].

Based on what has been reported and the controversies raised by other authors, this study aimed to characterize the radiological findings and patterns that are most important in the postacute phase and how these findings correlate with clinical and functional data in a small sample of patients who had COVID-19.

## 2. Materials and Methods

### 2.1. Study Design and Participants

The Research Ethics Committee approved this study of Pedro Ernesto University Hospital, State University of Rio de Janeiro, Rio de Janeiro, Brazil, under the number 31363230.1.0000.5282.

This was a retrospective study of 29 consecutively seen patients at the post-COVID-19 outpatient clinic of Piquet Carneiro Polyclinic and Pedro Ernesto University Hospital, State University of Rio de Janeiro, Rio de Janeiro, Brazil, between July and October 2021. All patients were diagnosed with COVID-19 using the polymerase chain reaction (PCR) test. The inclusion criteria were age> 18 and persistent respiratory symptoms after four weeks in nonhospitalized patients or patients discharged after hospitalization. The exclusion criteria were radiological tests with low technical quality and non-COVID-19 acute lung disease that could explain the respiratory symptoms.

Acute COVID-19 was classified as mild, moderate, or severe, according to the criteria established by Kamal et al. [16]. It was mild if the patients were isolated at home, moderate if oxygen therapy was necessary, and severe when admission to the intensive care unit (ICU) was essential.

### 2.2. Image Acquisition and Functional Tests

The CXRs were performed in an orthostatic position, with posteroanterior and left lateral views. The distance between the radiation source and the patient was 1.80 m. The equipment items used for image acquisition were the MULTIFIX *b* (Siemens) and Speed MF (Shimadzu) models.

Chest CT scans were performed using inspiratory, expiratory, and high-resolution reconstruction techniques. The 64-channel multidetector equipment was used (Brilliance 40, Philips Medical Systems, Cleveland, OH, USA), with a time of 4 s, current of 458 mA, a voltage of 120 kV, a thickness of two mm, an interval of one mm, and no use of iodinated contrast media.

The equipment used to perform the spirometry was the HD CPL (nSpire Health, Inc., Longmont, CO, USA). Before the test, patients rested for approximately 5–10 minutes. No smoking was necessary for 2 hours before the exam. Alcohol and coffee were not consumed for 4–6 hours before the test, and large meals were avoided for 1 hour before the test. During the test, patients remained seated, with their heads in a neutral position, and the nose clip was used.

The IOS was performed using an impulse oscillometer (Quark i2m, COSMED, Rome, Italy). During the IOS test, participants were instructed to remain seated, keeping their head in a neutral position, with manual support on the cheeks and the nostrils occluded by a clip, and then they would breathe normally for 40 seconds.

### 2.3. Image Interpretation

The CXR and CT images were analyzed using Horos software, version 3.3.6 (http//www.horosproject.org), on Dell workstations with 3-megapixel monitors. The technical parameters of the monitors were the factory defaults. The parenchyma and mediastinum windows were standardized at 1200 (width) and -600 (level) and 350 (width) and 50 (level), respectively. The radiographic and tomographic interpretations were conducted by consensus between two radiologists for CXR and another two for the CT scans. All of them were blinded to the clinical data. There was no statistical analysis of the agreement between the readers.

**Figure 1:**
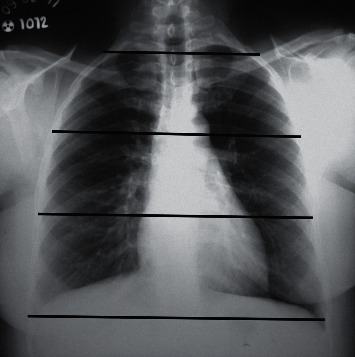
Posteroanterior CXR. Black lines demarcate the limits of the six zones.

For the radiographic examination, there was a semiquantitative evaluation of the extent of parenchymal involvement according to the method used by Litmanovich et al. [17] and Toussie et al. [18]. Involvement was classified as mild (2 zones), moderate (2–4 zones), or severe (>4 zones) (Figure 1).

For the tomographic findings of the extent of parenchymal involvement, a 15-point scale was implemented, on which 1–5 was considered mild, 6–10 moderate, and 11–15 severe.

The values obtained by spirometry were compared with the predicted values [19, 20]. The reference equations used were those of Pereira et al. [21], and all tests followed the standardization of the American Thoracic Society [22]. Regarding IOS, the following parameters were used: respiratory system resistance at 4 Hz (R4), 6 Hz (R6), 10 Hz (R10), and 20 Hz (R20); mean resistance between 4 and 20 Hz (Rm); resistance heterogeneity (R4-R20) [23]. Additionally, the area under the reactance curve (AX) was considered the resonance frequency [24].

### 2.4. Data Analysis

Statistical analysis was performed using SPSS software, version 26.

The normality of the distribution of the variables to characterize the sample was assessed using the Shapiro–Wilk test.

The following clinical variables were correlated with IOS, spirometry, CXR, and CT: age, gender, IMC, skin color, severity of acute COVID-19, hospitalization, ICU admission, oxygen therapy, mechanical ventilation, comorbidities, and smoking habit. The IOS variables studied were small airway disease and normal/abnormal tests. The spirometry variable analyzed was the functional status (normal or restrictive/obstructive). The CXR variables analyzed were the CXR extent of impairment (normal/mild and moderate/severe), lack of definition of the bronchovascular markings, and architectural distortion. The CT variables analyzed were the CT extent of impairment (normal/mild and moderate/severe), presence of air trapping, and tomographic patterns (normal, reabsorption, airway disease, fibrosis-like lesions, lesions similar to nonspecific interstitial pneumonia [NSIP], lesions similar to OP, and mixed).

The interrelationships between the clinical variables and IOS, spirometry, CXR, and CT and between the imaging and functional studies were analyzed using the chi-square (*χ*^2^) or Fisher's exact test. The significance level adopted was lower than 5%.

## 3. Results

Of the 29 patients studied, 19 were women (65.5%). Regarding skin color, 18 were brown/black (62.1%) and 11 were white (37.9%). The mean age was 60 ± 13 years (35–88 years). The mean body mass index (kg/m^2^) was 31.0 ± 4.8.

Regarding disease severity in the acute phase, eight patients had mild disease (27.6%), 15 had moderate disease (61.7%), and six had severe disease (20.7%). In the sample, 18 patients (62.1%) were hospitalized. The most frequent symptoms related to postacute COVID-19 were dyspnoea and fatigue (82.4%) and cough (42.3%). Comorbidities were present in 23 patients (79.3%), with the most frequent hypertension (58.6%) and diabetes mellitus (44.8%). Only eight patients were smokers (27.6%). The mean time interval between the onset of the disease and radiological examinations was 8.8 months.

Abnormal spirometry findings were evident in 13 patients (44.8%): 12 with a restrictive pattern and one with an obstructive pattern. Of the 24 patients who underwent IOS, 18 (75%) had abnormal results. Among the patients with abnormal IOS results, 12 (50%) had abnormal alterations compatible with small airway disease. The predominance of altered spirometry was associated with moderate/severe CT involvement (*p* = 0.017; 69.2%, CI 95%: 44.1%–94.3%) (Table 1), which indicates that of the 13 cases with moderate/severe CT, 9 (69.2%) had altered spirometry. There was no significant association between IOS and tomographic or radiographic parameters.

Regarding the severity of the extent of CT impairment, 14 cases were considered mild (48.3%), eight were moderate (27.6%), and five were severe (17.2%), while two cases had no changes (6.9%). The most frequent findings on CT were GGO (86.2%), septal thickening (62.1%), air trapping (51.7%), parenchymal bands (55.2%), bronchiectasis (41.4%), peripheral vascular ectasia (34.5%), subpleural lines (34.5%), volumetric reduction (13.8%), signs of bronchiolitis (10.3%), crazy paving (10.3%), signs of peripheral arterial hypertension (10.3%), emphysema (6.9%), and consolidation suggestive of OP (6.9%).

**Figure 2:**
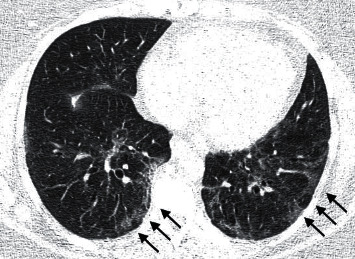
Chest CT in a lung window. An axial section at the level of the lower lobes. Subpleural ground-glass opacities (arrows).

**Figure 3:**
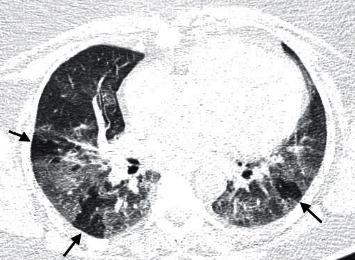
Chest CT in a lung window. Expiratory apnoea. An axial section at the level of the lower lobes highlights areas of air trapping (arrows).

**Figure 4:**
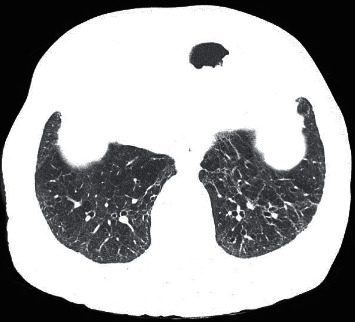
Chest CT in a lung window. An axial section at the level of the lower lobes highlights parenchymal bands and bronchiectasis. Fibrosis-like lesions pattern.

The patterns found on CT were resorption (79.3%) (Figure 2), airway disease (66.5%) (Figure 3), mixed (several concomitant patterns) (51.7%), fibrosis-like lesions (37.9%) (Figure 4), lesions similar to NSIP (13.8%), and lesions similar to OP (6.9%). In total, 6.9% of the cases had normal tests. Air trapping was significantly associated with the airway disease pattern (*p* = 0.0001; 100%, CI 95%: 100%–100%), which indicates that all 15 cases with air trapping had an airway disease pattern. There was a significant association between the classifications of moderate/severe and normal/mild patterns on CT and CXR (*p* = 0.003; 93.3%-CI 95%: 77.8%–100%) (Table 1). Of the 13 cases with moderate/severe CT, 12 (93.3%) had abnormal CXR.

**Figure 5:**
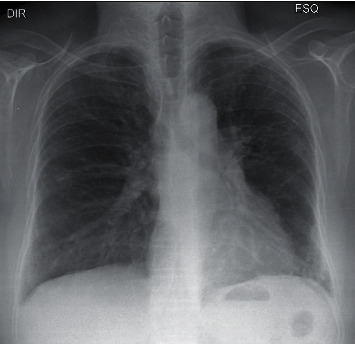
Posteroanterior CXR. Multifocal bronchovascular lack of definition.

Regarding the severity of the extent of CXR impairment, three cases were considered mild (10.3%), ten were moderate (34.5%), and eight were severe (27.6%), while eight cases had no changes (27.6%). The most frequent findings on CXR were lack of definition of the bronchovascular pattern (72.4%) (Figure 5), volume reduction (6.9%), architectural distortion (6.9%), hyperinflation (3.4%), and pleural thickening/effusion (3.4%).

Patients with moderate/severe impairment on CXR were associated with a higher frequency of hospitalization (*p* = 0.033; 77.8%, CI 95%: 58.6%–97.0%). This expresses that of the 18 cases with abnormal CXR, 14 (77.8%) were hospitalized, and they were also associated with moderate/severe clinical classifications in the acute phase (*p* = 0,017; 88.9%, CI 95%: 74.4%–100%). This expresses that of the 18 cases with abnormal CXR, 16 (88.9%) had a moderate/severe clinical grade (Table 2).

## 4. Discussion

To our knowledge, this is the first study on postacute COVID-19 that correlates clinical data with CXR, CT, spirometry, and IOS tests. The main goals of this study are to emphasize postacute CT patterns other than fibrosis, such as the resorption pattern, and to highlight CXR as a tool aligned with the events of the acute phase and the degree of long-term impairment.

The epidemiological profile of patients is similar to that found in other studies on COVID-19 [25, 26]. However, our sample's median follow-up time (8.8 months) denotes quite prolonged clinical pictures of the disease, which shows two things: postacute COVID-19 manifestations can remain relevant for a long time and the ease of access to the health system in Brazil may not be ideal.

The most frequent symptoms, namely, dyspnoea and fatigue, are consistent with those reported in the literature [6, 27–30]. Regarding spirometry, a high percentage of patients showed changes (44.8%), with a predominance of the restrictive pattern. This is not in line with the study by Moreno-Pérez et al. [8], in which only 9.3% of the exams showed alterations, with a predominance of the obstructive pattern. The predominance of restriction in our study makes sense because CT showed lesion patterns similar to fibrosis and NSIP in 37.9% and 13.8% of cases, respectively. This may be associated with a lower diffusion capacity [27]. In addition, there was an association between moderate/severe impairment on CT and altered spirometry, which reflects the ability of both tests to detect the involvement burden of the lung interstitium due to the disease.

Despite the number of altered IOS tests and CT scans, there was no statistical association between the IOS data and spirometry or CT. This finding can be explained by the type of assessment performed by IOS, which involves pulmonary and extrapulmonary structures. Lopes et al. [15] reported a weak association between spirometry and IOS data in a sample of 117 patients with postacute COVID-19.

The altered IOS tests for small airway disease (51.7%) and the presence of air trapping on CT (50%) may represent one of two phenomena: constrictive bronchiolitis or a primary abnormality of the pulmonary microvasculature (endotheliitis and microthrombosis). Both alterations can produce a ventilation/perfusion mismatch [14, 31–33]. Furthermore, the finding of 34.5% of peripheral vascular ectasia on CT, a common phenomenon in the acute phase, represents a persistent microvascular impairment frequently associated with GGO. From a cellular point of view, CD8 lymphocytes that respond in an exacerbated or dysregulated manner are associated with the inflammatory and fibrotic changes of COVID-19, including small airway disease [28, 34]. Other studies have also cited small airway involvement in postacute COVID-19 [10, 11, 29, 32]. Cho et al., like us, found no association between air trapping and obstructive patterns in spirometry [14]. Most likely, less than 75% of the small airways are compromised; what would be the minimum value to be detected by spirometry as an obstructive pattern.

There was no association between comorbidities and the degree of CT and CXR impairments. Contradictorily, comorbidities were more relevant in the CXR normal/mild group. The low number of patients may explain these findings, and additional studies with a larger sample are needed to reach more consistent conclusions.

The most frequent tomographic findings, especially GGO, are in line with other studies [10, 11, 35–39]. GGO is the main component of the resorption pattern and was the most frequent component in our sample. This resorption GGO is different from the same lesion found in the acute phase. Postacute GGO reflects the ongoing residual inflammatory process and tends to disappear over time.

In line with other studies [40, 41], fibrosis-like lesions were prevalent in our sample, which may be secondary to deranged pulmonary immune function [33]. However, the distribution of tomographic patterns shows that it is inadequate to focus the discussion of follow-up exclusively on the existence and longevity of lesions similar to fibrosis. These patterns often overlap, and pulmonary symptoms should reflect the interaction of several pathophysiological phenomena. Furthermore, it is necessary to monitor these patients for more extended periods. Recently, several studies presented follow-ups more than a year after the acute event, and 54% of the patients had tomographic alterations in this period [42, 43].

There was an association between CXR and CT severity classifications. In addition, there was an association between the moderate/severe CXR classification and two clinical parameters, hospitalization and moderate/severe clinical classification, in the acute phase of COVID-19. This is in line with some other studies [11, 12]. Therefore, despite the lower sensitivity of CXR compared to CT [11], CXR may be used as an initial test for the detection and monitoring of postacute complications, which corroborates the guidelines of the British Thoracic Society [9]. It is also worth noting that the most frequent radiographic alteration, the lack of definition of the bronchovascular pattern, is a nonspecific finding. It may represent GGO, fibrosis-like lesions, and bronchiectasis. Therefore, when it is essential to define a specific lung pattern, CT can complement CXR.

Some limitations of this research should be pointed out. Because this is a retrospective study, we evaluated patients with different follow-up times of postacute COVID-19. Although the IOS was performed at a time similar to that of other exams, it was not performed on the same day as the CXR, CT, and spirometry. We do not have the vaccination history of the patients, which would be important to correlate with radiological and functional data. Finally, the small sample size of this preliminary research reinforces the need for future studies that incorporate a larger number of patients.

## 5. Conclusions

In conclusion, the results of this study show that CXR is a relevant examination and may be used to detect nonspecific alterations during the follow-up of post-COVID-19 patients. Furthermore, small airway disease is an essential finding in postacute COVID-19 syndrome, and we postulate a connection between this pattern and the persistently low-level inflammatory state of the lung.

## Figures and Tables

**Table 1:** Correlation of several variables with the extent of CT impairment^*∗*^.

Variable	Extent of CT impairment				*p-value*
	Moderate/severe		Normal/mild		
	*n*	%	*n*	%	
Age (median)					
<62 years	5	38.5	9	56.3	0.34
≥62 years	8	61.5	7	43.8	
Gender					
Masculine	6	46.2	4	25.0	0.21
Feminine	7	53.8	12	75.0	
Skin color					
White	5	38.5	6	37.5	0.63
Brown/black	8	61.5	10	62.5	
Disease severity (acute phase)					
Mild	2	15.4	6	37.5	0.49
Moderate	8	61.5	7	43.8	
Severe	3	23.1	3	18.8	
Hospitalization					
Yes	10	76.9	8	50.0	0.13
No	3	23.1	8	50.0	
Comorbidities					
Yes	9	69.2	14	87.5	0.23
No	4	30.8	2	12.5	
CXR impairment extent					
Moderate/severe	12	**92.3**	6	**37.5**	**0.003**
Normal/mild	1	7.7	10	62.5	
Spirometry					
Normal	4	30.8	12	75.0	**0.017**
Restrictive/obstructive	9	**69.2**	4	**25.0**	

*∗*Chi-square or Fischer's exact test. CT = computed tomography; CXR = chest radiograph.

**Table 2:** Correlation of several variables with the extent of CXR impairment^*∗*^.

Variable	Extent of CXR impairment				*p-value*
	Moderate/severe		Normal/mild		
	*n*	%	*n*	%	
Age (median)					
<62 years	7	38.9	7	63.6	0.20
≥62 years	11	61.1	4	36.4	
Gender					
Masculine	7	38.9	3	27.3	0.41
Feminine	11	61.1	8	72.7	
Skin color					
White	7	38.9	4	36.4	0.60
Brown/black	11	61.1	7	63.6	
Disease severity (acute phase)					
Mild	2	11.1	6	54.5	**0.017**
Moderate/severe	16	88.9	4	36.4	
Hospitalization					
Yes	14	**77.8**	4	**36.4**	**0.033**
No	4	22.2	7	63.6	
Comorbidities					
Yes	12	**66.7**	11	**100**	**0.039**
No	6	33.3	0	0	
Spirometry					
Normal	8	44.4	8	72.7	0.13
Restrictive/obstructive	10	55.6	3	27.3	

*∗* Chi-square or Fischer's exact test. CXR = chest radiograph.

## Data Availability

The data used to support the findings of this study are available from the corresponding author upon reasonable request.
